# Pretreatment feeding‑stoma placement in advanced esophageal cancer: defining optimal patient selection criteria

**DOI:** 10.1007/s10388-026-01210-6

**Published:** 2026-05-21

**Authors:** Hsueh-Chien Chiang, Chien-Ming Chiang, Ying-Yuan Chen, Shang-Yin Wu, Forn-Chia Lin, Tzu-Hui Pao, Ta-Jung Chung, Bor-Shyang Sheu, Wei-Lun Chang

**Affiliations:** 1 Department of Internal Medicine, College of Medicine, National Cheng Kung University Hospital, National Cheng Kung University, No.138, Sheng Li Road, Tainan, 704 Taiwan; 2 Institute of Clinical Medicine, College of Medicine, National Cheng Kung University Hospital, National Cheng Kung University, Tainan, Taiwan; 3 Department of Surgery, College of Medicine, National Cheng Kung University Hospital, National Cheng Kung University, Tainan, Taiwan; 4 Department of Oncology, College of Medicine, National Cheng Kung University Hospital, National Cheng Kung University, Tainan, Taiwan; 5 Department of Radiology, College of Medicine, National Cheng Kung University Hospital, National Cheng Kung University, Tainan, Taiwan

**Keywords:** Esophageal squamous cell carcinoma, Feeding-stoma, Tumor-occupying proportion, Cachexia, Chemoradiation

## Abstract

**Background:**

The indication of feeding-stoma creation (gastrostomy/jejunostomy) in esophageal squamous cell carcinoma (ESCC) patients and its impact on survival remain unclear. We aimed to identify patient subgroups who benefit from feeding-stoma placement before concurrent chemoradiation therapy (CCRT).

**Methods:**

We did a secondary analysis of prospective cohort. A total of 260 patients with advanced ESCC who underwent CCRT between April 2008 and March 2024 were included. Tumor-occupying proportion (tumor area/total lumen area) and other tumor characteristics were measured from standardized endoscopic images. Characteristics that predict post-CCRT cachexia was identified and validated as indications of feeding-stoma creation.

**Results:**

Post-CCRT cachexia developed in 60.7% of patients. Independent predictors of cachexia included longer tumor length and bigger tumor-occupying proportion. ROC curve found tumor length ≥ 6 cm (area under ROC (AUC): 0.760, *P* < 0.001) and tumor-occupying proportion ≥ 70% are optimal to predict post-CCRT cachexia (AUC: 0.620, *P* = 0.001). Sixty-two patients (23.8%) underwent pretreatment feeding-stoma creation. In the overall cohort, feeding-stoma creation was not associated with a longer progression-free survival (PFS) or overall survival (OS). In patients with tumor-occupying proportion ≥ 70%, feeding-stoma creation was associated with reduced cachexia incidence, better treatment response (OR: 2.78, *P* = 0.028), longer median PFS (6 vs. 4 months, *P* = 0.012) and OS (11 vs. 9 months, *P* = 0.009). In contrast, feeding-stoma creation was not associated with an improved treatment response or survival in patients with tumor-occupying proportion < 70% or in patients with different tumor length.

**Conclusion:**

Tumor-occupying proportion is a novel metric that predicts cachexia and survival in advanced ESCC. Pretreatment feeding-stoma creation was associated with a better treatment response and longer survival only in patients with tumor-occupying proportion ≥ 70%, supporting its use as a selection criterion for this invasive procedure.

**Graphical abstract:**

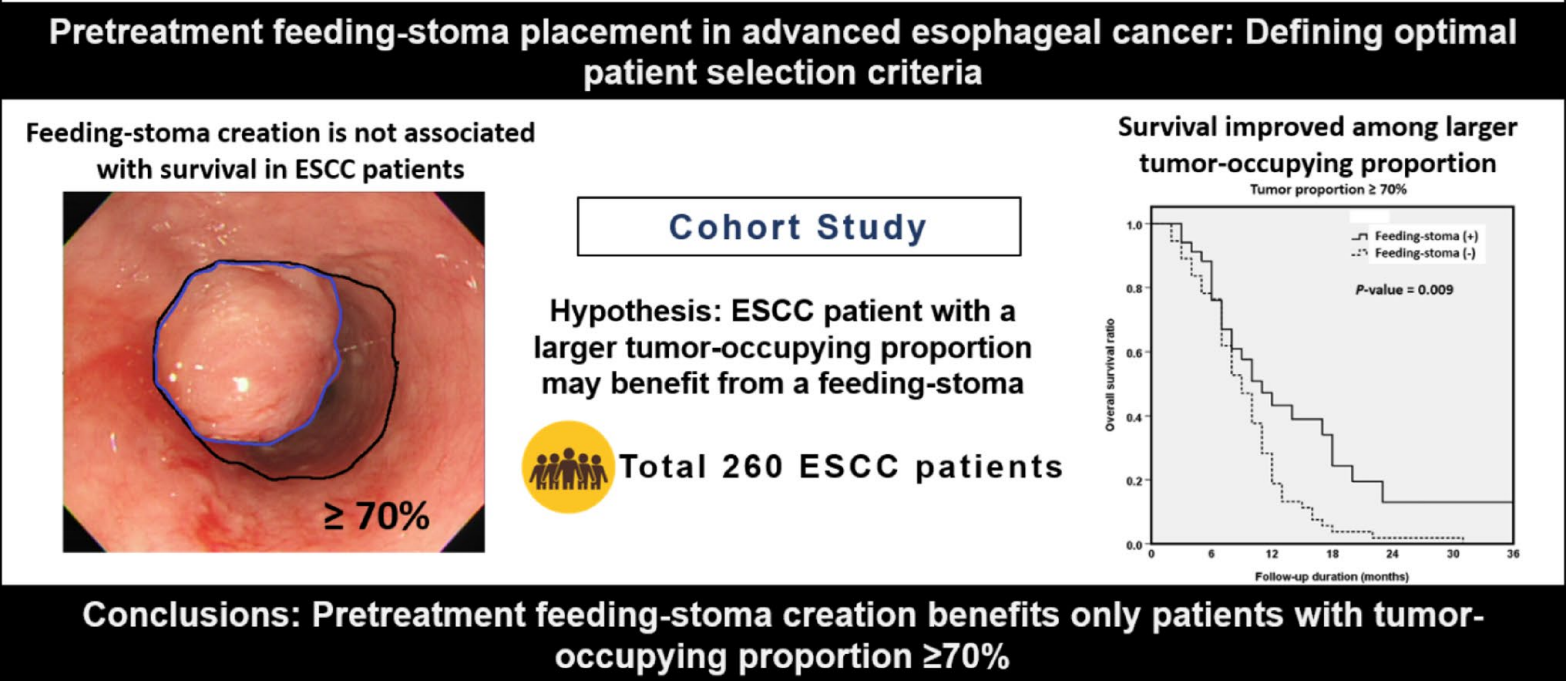

**Supplementary Information:**

The online version contains supplementary material available at 10.1007/s10388-026-01210-6.

## Introduction

Esophageal cancer ranks among the most prevalent gastrointestinal cancers globally, with approximately 473,000 new cases diagnosed each year [1]. The predominant histological type is squamous cell carcinoma (SCC), which exhibits a significantly higher incidence in Eastern countries compared to Western nations [2]. Most patients with esophageal SCC (ESCC) are diagnosed at advanced stages, and their initial treatment typically involves concurrent chemoradiation therapy (CCRT), which may be followed by esophagectomy in selected cases [2]. Unfortunately, the prognosis for ESCC remains grim, with a 5-year survival rate of less than 20%[2].

Dysphagia is the most prevalent symptom of esophageal cancer, typically starting with difficulty swallowing solid foods and progressing to liquids as the tumor grows and narrows the esophagus [3]. Following dysphagia, significant weight loss is common due to difficulties in eating [3, 4]. Such weight loss is closely linked to malnutrition, a well-recognized factor that adversely affects outcomes of cancer patients, including those with esophageal cancer [4, 5]. Patients with esophageal cancer are particularly vulnerable to malnutrition due to tumor-induced esophageal obstruction. Reports indicate that nearly 80% of esophageal cancer patients experience malnutrition, significantly limiting their treatment options and effectiveness [6, 7]. Malnutrition leads to immune dysfunction, diminished performance status, increased treatment-related complications, and elevated mortality rates [8, 9].

Feeding-stoma creation such as gastrostomy and jejunostomy play a crucial role in managing the nutritional needs of patients with esophageal cancer, particularly for those patients with dysphagia [10]. The primary advantage of gastrostomy and jejunostomy is its ability to provide stable nutritional support, allowing higher energy feeds and shortening the length of hospital stay compared to nasogastric tubes [11, 12]. Improved nutritional status has been linked to better tolerance of therapies and overall survival of cancer patients [13]. Recent research demonstrated that gastrostomy and jejunostomy before definite CCRT are associated with better nutritional status in unresectable locally advanced esophageal cancer patients [14].

However, the pretreatment gastrostomy and jejunostomy procedures have not demonstrated a significant improvement in overall survival time for ESCC patients [14]. Furthermore, some patients may suffer from the adverse events of the feeding tubes, such as infections, dislodgement, leakage, and increased healthcare visits [15, 16]. This raises critical questions about the appropriateness of recommending these interventions for all such patients prior to CCRT. It appears that only those individuals with a bulky esophageal tumor and solid food dysphagia may derive meaningful benefits from gastrostomy or jejunostomy.

This study aims to delineate the specific patient population within the cohort of advanced esophageal cancer who may experience survival benefits from pre-CCRT feeding-stoma creation. Identification of this population will facilitate more tailored interventions to improve outcomes in this challenging clinical scenario.

## Methods

### Study design and setting

An observational cohort study was conducted in a tertiary referral medical center (National Cheng Kung University Hospital, Tainan, Taiwan). The study period spanned April 2008 to March 2024. The protocol was approved by the Institutional Review Board of the National Cheng Kung University Hospital (B-BR-106-082).

### Participants

Eligible patients had histologically confirmed, advanced ESCC, the predominant histological type in Taiwan (~ 91%). Patients were excluded if they (1) died during or within 1 month after completion of CCRT, or (2) lacked baseline endoscopic or computed tomography (CT) imaging required for tumor measurement and staging. Patients were recruited consecutively from institutional cancer registries.

### Treatment and follow-up

All patients received baseline workup including endoscopic ultrasound (EUS), chest CT, and FDG positron emission tomography (PET)/CT scan (optional) according to NCCN guidelines [17]. Before treatment, feeding-stoma was created based on discussions between physician and patient, with the type selected accordingly. For patients who underwent gastrostomy, enteral feeding commenced 1 day postoperatively, with the calorie goal gradually advanced to 20–30 kcal/kg/day by increasing 300 kcal every 2 days. For those who underwent jejunostomy, feeding started 2 days postoperatively, advancing to the same calorie goal with same speed. Oral intake is allowed if dysphagia improved. If oral intake was adequate following CCRT, use of the feeding-stoma could be discontinued.

CCRT course consisted of intravenous platinum-based chemotherapy with concurrent radiotherapy to the primary tumor and regional lymph nodes. Post-treatment evaluation with EUS and CT was performed at 4 to 6 weeks after CCRT. Patients were followed every 3 months for at least 2 years or until death, with surveillance for disease progression and survival outcomes.

### Definition of cancer cachexia and other variables

Body weight was measured at baseline and at post-CCRT EUS examination using the same calibrated weighing scale. All patients underwent overnight fasting and bladder emptying before measurement. Cachexia was defined as weight loss of > 5% or > 2% with a body mass index (BMI) < 20 kg/m^2^ after CCRT completion [18, 19]. Dysphagia severity was graded using the Mellow and Pinkas Dysphagia Scoring System (0: normal, 1: able to eat everything but with some difficulty, 2: dysphagia to solids, 3: dysphagia to semi-solid, 4: dysphagia to fluids) [20].

### Outcome measurement

The treatment response was evaluated by both EUS and chest CT scans [21]. In EUS, a miniprobe ultrasound catheter (20 MHz, Olympus Corp.) was applied to measure the post-CCRT esophageal wall thickness. The follow-up CT scan was compared with the baseline scan that was done before the initiation of therapy to define the treatment response of metastatic tumor foci. Treatment responses were classified as CR (complete response, no residual tumor), PR (partial response, ≥ 30% decrease in the tumor thickness from baseline), PD (progressive disease, ≥ 20% increase in the tumor thickness compared to the nadir or development of new metastatic lesions), or SD (stable disease, neither sufficient shrinkage for PR nor sufficient increase for PD). In this study, CR or PR were defined as good treatment response and those of SD or PD were poor treatment response.

The primary endpoint of our study is the overall survival (OS). Secondary endpoints include progression-free survival (PFS), and post-CCRT cachexia. OS was defined as the interval from treatment initiation to death from any cause. Progression-PFS was also measured, which was defined as the interval from treatment initiation to tumor progression or death.

### Measurement of tumor-occupying proportion and other tumor features

**Fig. 1:**
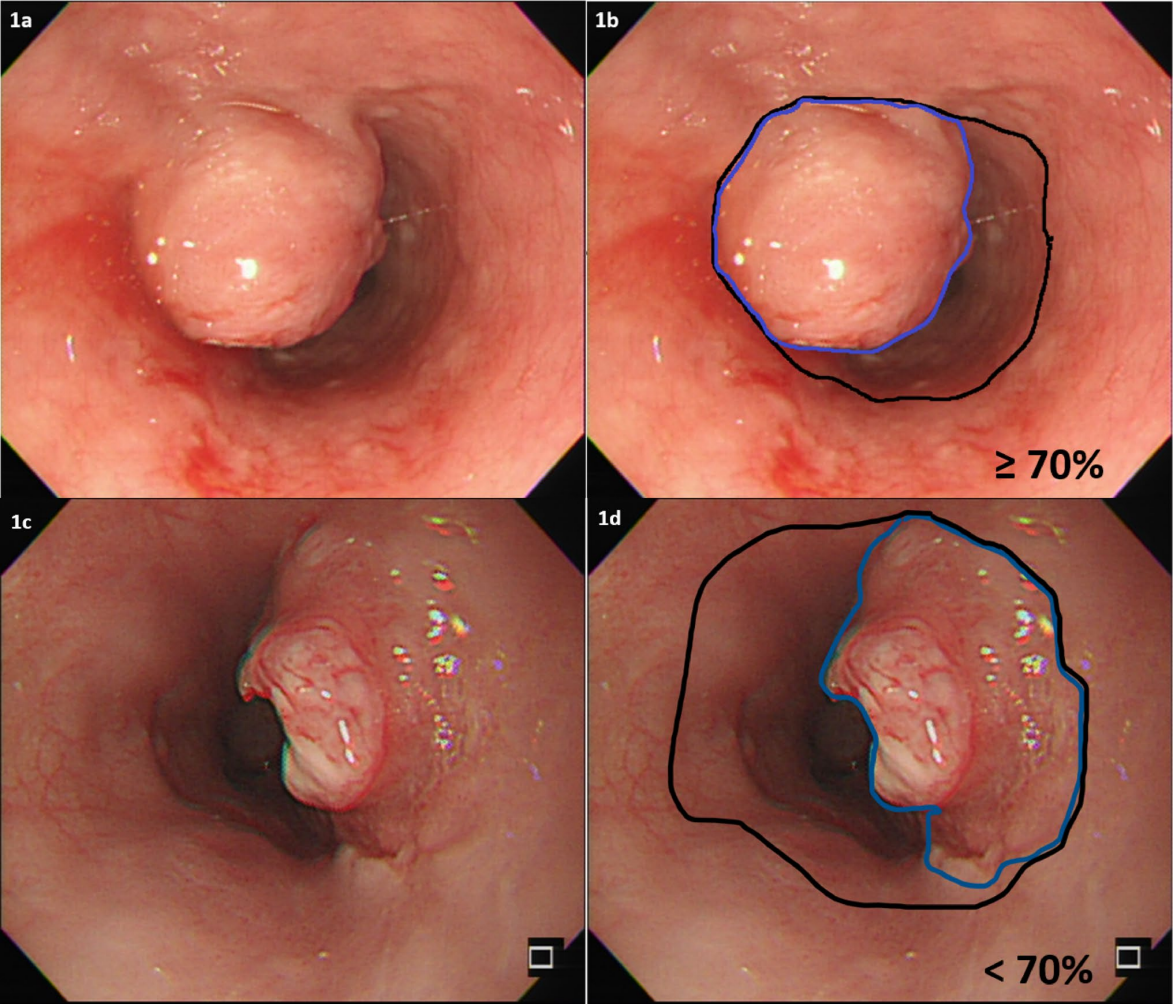
Measurement of tumor-occupying proportion. **a**, **c** Endoscopic view of the esophageal tumor. **b**, **d** The blue line delineates the tumor area, and the black line delineates the total esophageal lumen area at the same level. The tumor-occupying proportion was calculated by dividing the tumor area by the total lumen area

To objectively assess the extent of esophageal lumen obstruction, we developed a novel metric, the *tumor-occupying proportion* (tumor area/total lumen area), calculated from high-quality endoscopic images (Olympus CV-260 or CV-290) obtained under standardized conditions. Two blinded investigators (H-C Chiang and C-M Chiang) independently reviewed all captured frames and selected the clearest image that simultaneously displayed both the largest tumor and the surrounding normal lumen in the same vertical plane, providing the most en face view of the lumen. This standardized selection method ensured consistent image orientation and accurate assessment of tumor-occupying proportion. Using ImageJ’s selection tools, the visible lumen and the tumor boundaries within the same vertical plane were manually delineated. The tumor-occupying proportion was then calculated as the ratio of the tumor area to the total esophageal lumen area (Fig. 1). Inter-observer reliability was assessed by intraclass correlation coefficient (ICC).

Tumor length and thickness were measured according to a standardized protocol described previously [21]. Esophageal patency was defined as the ability to pass an upper endoscope (GIF-CV290, Olympus Corp.) [21].

### Statistical analysis

Continuous variables were compared using Student’s t-test or Mann-Whitney U test, and categorical variables using Pearson’s χ2 test or Fisher’s exact test, as appropriate. Logistic regression was used to identify predictors of cachexia. Variables with *p* < 0.2 in univariate analysis were included in a multivariate model. Survival differences between patient groups were assessed using Kaplan-Meier curve and compared with the log-rank test. Propensity score matching (PSM), as a sensitivity analysis, was used to compare the outcomes of the creation of feeding-stoma in subgroup analysis to minimize potential bias. A propensity score was generated via the nearest matching algorithm with a 1:1 ratio for PSM to balance pre-treatment variables (age, gender, tumor overall stage, tumor length, and tumor depth), and pairs of patients were matched with a tolerance of 0.02 without replacement. All tests were two-tailed, with *p* < 0.05 considered statistically significant. All statistical analyses were performed using SPSS (version 25.0; IBM Corporation, Armonk, NY, USA).

## Results

### Patient characteristics

Between April 2008 and March 2024, 268 patients with histologically confirmed advanced ESCC who received CCRT were screened. Five patients were excluded due to missing pretreatment endoscopy or CT imaging data, and three patients died before the CCRT completion, leaving 260 eligible patients for analysis. All enrolled patients completed CCRT and had follow-up data available for cachexia and survival analysis. Among them, 249 (95.7%) were male with a mean age of 57.9 ± 9.9 years (Table 1). The mean pretreatment BMI was 21.9 ± 3.6 kg/m^2^. Mean serum albumin, prealbumin, and transferrin were 4.1 ± 0.4 g/dL, 21.5 ± 7.1 mg/dL, and 218 ± 53 mg/dL, respectively. Mean Tumor length, thickness, and occupying proportion were 5.7 ± 2.7 cm, 1.55 ± 0.89 cm, and 58.8 ± 22.2%, respectively. The distribution of tumor-occupying proportion is listed in Table 1. The inter-observer reliability for tumor-occupying proportion measurements was excellent (ICC = 0.899, *p* < 0.001). The relationship between tumor-occupying proportion with T stage and dysphagia score is shown in Supplementary Fig. 4.

**Table 1:** Baseline characteristics before definitive CCRT (*N* = 260)

Characteristics	*N* = 260
Age (years)	57.9 ± 9.9
Gender (M: F)	249: 11
Body weight (kg)	60.2 ± 11.2
BMI (kg/m^2^) *	21.9 ± 3.6
Drinking history (%)	174 (66.9)
Smoking history (%)	192 (73.8)
Betel nut history (%)	130 (50)
Albumin (g/dL)	4.1 ± 0.4
Prealbumin (mg/dL)	21.5 ± 7.1
Transferrin (mg/dL)	218.2 ± 53.6
Dysphagia score (%)	
0	67 (25.7)
1	34 (13.0)
2	116 (44.6)
3	32 (12.3)
4	11 (4.2)
Cancer stage ^‡^ (%)	
Stage I	6 (2.3)
Stage II	15 (5.7)
Stage III	83 (31.9)
Stage IV	156 (60)
T stage- T1: T2: T3 (%)	
T1	13 (5.0)
T2	21 (8.0)
T3	226 (86.9)
*Endoscopy findings*	
Tumor location (%)	
Upper third	64 (24.6)
Middle third	72 (27.6)
Lower third	124 (47.6)
Tumor length (cm)	5.7 ± 2.6
Tumor thickness (cm)	1.9 ± 1.0
Tumor-occupying proportion^α^ (%)	
0–10%	3 (1.1)
11–20%	8 (3.0)
21–30%	24 (9.2)
31–40%	27 (10.3)
41–50%	29 (11.1)
51–60%	28 (10.7)
61–70%	51 (19.6)
71–80%	46 (17.6)
81–90%	30 (11.5)
91–100%	14 (5.3)
Esophageal patency (%) ^†^	190 (73.0)

*Body mass index (BMI) is calculated by dividing an adult’s weight in kilograms by their height in meters squared

^‡^Cancer staging based on AJCC 7th version

^α^The tumor-occupying proportion was calculated as the ratio of the tumor area to the total esophageal lumen area

^†^Esophageal patency was defined as the ability to successfully pass an upper endoscope (GIF-CV290, Olympus Corp.) through the esophagus

After completion of CCRT, 24 patients (9.2%) had complete remission, 126 patients (48.4%) had partial response, 53 patients (20.3%) were stable disease, and 57 patients (21.9%) had progressive disease. The median follow-up time for survivors of the entire cohort was 16 months (interquartile range: 12–26 months).

### Factors associated with post-CCRT cachexia

Post-CCRT cachexia occurred in 158 patients (60.7%). Table 2 presents factors associated with post-CCRT cachexia. Univariate logistic regression analysis identified lower baseline prealbumin levels, higher cancer stage, longer tumor length, and greater tumor-occupying proportion as significant factors. Multivariate analysis confirmed that longer tumor length (OR:1.23, 95% CI: 1.04–1.45, *P* = 0.013) and higher tumor-occupying proportion (OR:1.03, 95% CI: 1.00-1.06, *P* = 0.035) were independent predictors of post-CCRT cachexia.

**Table 2:** Logistic regression analysis of factors associated with post-CCRT cachexia (*N* = 260)

Factors	Univariate		Multivariate	
	OR (95% CI)	*p*	OR (95% CI)	*p*
Age (years)	0.99 (0.97–1.02)	0.764		
Gender (male)	1.90 (0.49–7.29)	0.345		
BMI (kg/m^2^)	1.06 (0.98–1.14)	0.118	1.07 (0.97–1.18)	0.173
Albumin (g/dL)	0.69 (0.36–1.33)	0.275		
Prealbumin (mg/dL)	0.95 (0.91–0.99)	0.040	0.97 (0.92–1.02)	0.278
Transferrin (mg/dL)	1.00 (0.99-1.00)	0.515		
Dysphagia score	1.33 (1.06–1.67)	0.013	0.92 (0.61–1.38)	0.707
Cancer stage	1.61 (1.07–2.42)	0.022	1.10 (0.58–2.07)	0.758
Endoscopy findings				
Tumor length (cm)	1.17 (1.04–1.31)	0.007	1.23 (1.04–1.45)	0.013
Tumor thickness (cm)	1.47 (0.89–2.43)	0.127	0.95 (0.65–1.41)	0.829
Tumor-occupying proportion * (%)	1.02 (1.01–1.03)	< 0.001	1.03 (1.00-1.06)	0.035
Esophageal patency ^†^	1.62 (0.88–2.98)	0.115	0.99 (0.31–3.17)	0.999
Feeding-stoma (+)	1.07 (0.59–1.93)	0.813		

*The tumor-occupying proportion was calculated as the ratio of the tumor area to the total esophageal lumen area

^†^Esophageal patency was defined as the ability to successfully pass an upper endoscope (GIF-CV290, Olympus Corp.) through the esophagus

### Optimal cut-off values for predicting post-CCRT cachexia

**Fig. 2:**
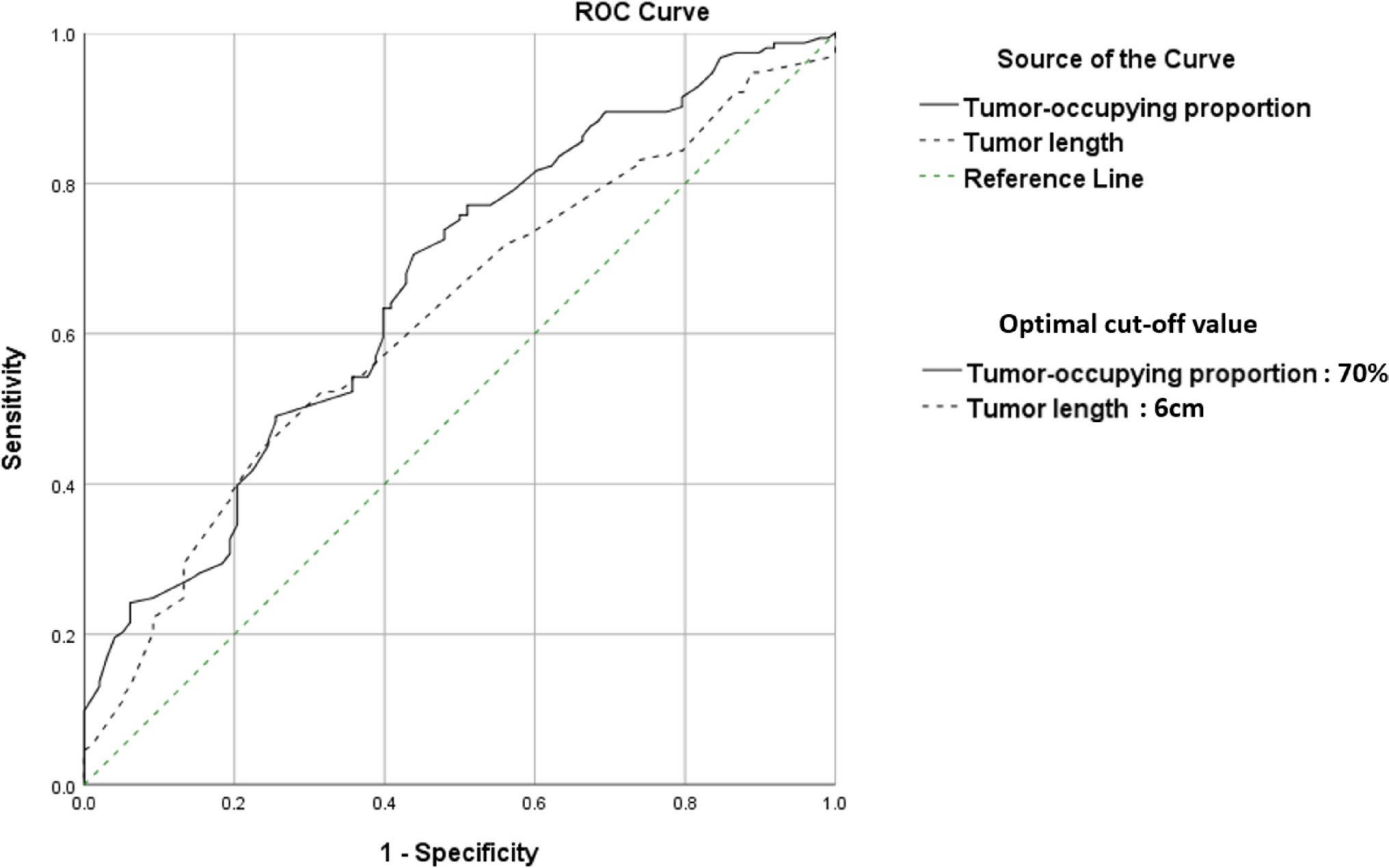
Receiver operating characteristic curves of factors predicting post‑treatment cachexia. The curves illustrate the predictive performance of tumor-occupying proportion and tumor length

**Fig. 3:**
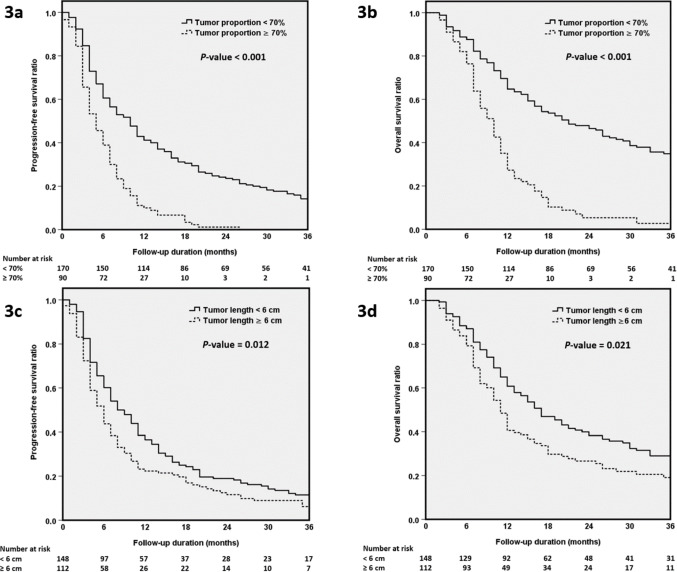
Comparison of survival in patients with different tumor‑occupying proportion and tumor length. **a** Comparison of PFS in patients with different tumor-occupying proportion. **b** Comparison of OS in patients with different tumor-occupying proportion. **c** Comparison of PFS in patients with different tumor length. **d** Comparison of OS in patients with different tumor length

Using ROC curve analysis and the Youden index, we identified a tumor-occupying proportion of 70% as the optimal cut-off for predicting post-CCRT cachexia (area under the ROC curve (AUC): 0.760, *P* < 0.001; Fig. 2). Similarly, a tumor length of 6 cm was determined to be the optimal threshold for predicting post-CCRT cachexia (AUC: 0.620, *P* = 0.001; Fig. 2). Kaplan-Meier survival analysis demonstrated significantly longer PFS (10 vs. 5 months, *P* < 0.001; Fig. 3a) and OS (21 vs. 10 months, *P* < 0.001; Fig. 3b) in ESCC patients with a tumor-occupying proportion < 70%, compared to those with ≥ 70%. Similarly, patients with a tumor length < 6 cm exhibited longer PFS (8 vs. 6 months, *P* = 0.012; Fig. 3c) and OS (17 vs. 11 months, *P* = 0.021; Fig. 3d) than those with tumor length ≥ 6 cm. Notably, patients with a tumor-occupying proportion ≥ 70% had lower serum prealbumin levels, higher dysphagia score, more advanced T-stage disease, and greater tumor length (Supplementary Tables 1 & supplementary Fig. 4). In parallel, those with tumor length ≥ 6 cm demonstrated more advanced T-stage and N-stage disease, as well as a higher tumor-occupying portion (Supplementary Table 2).

### Identifying the patients with a better outcome from feeding‑stoma creation

Among 260 ESCC patients, 62 patients (23.8%) underwent feeding-stoma creation before CCRT (24 with gastrostomy and 38 with jejunostomy). Pretreatment feeding-stoma creation was not significantly associated with a reduced incidence of cachexia (*P* = 0.813, Table 2) or improved treatment response across the entire cohort (*P* = 0.155, Supplementary Table 3). Kaplan-Meier survival analysis showed no PFS (Supplementary Fig. 1a) or OS (Supplementary Fig. 1b) benefit from feeding-stoma creation in all patients with advanced ESCC.

**Fig. 4:**
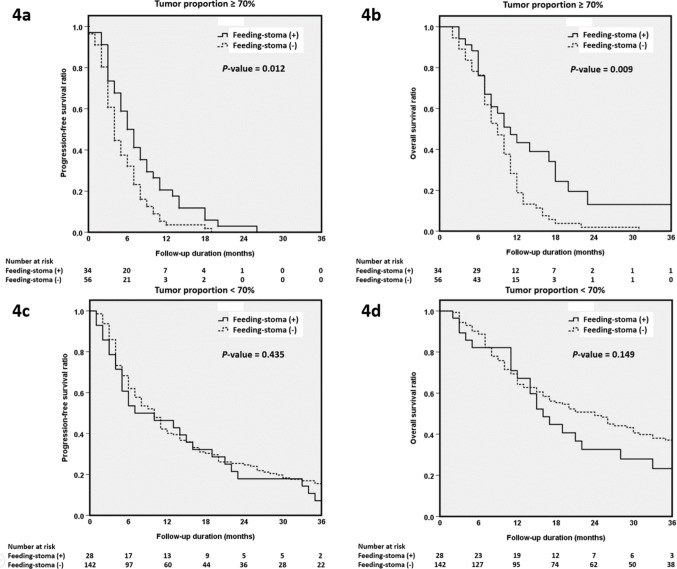
Comparison of survival between patient with and without feeding‑stoma creation, stratified by tumor‑occupying proportion. **a** PFS in patients with tumor-occupying proportion ≥ 70%. **b** OS in patients with tumor-occupying proportion ≥ 70%. **c** PFS in patients with tumor-occupying proportion < 70%. **d** OS in patients with tumor-occupying proportion < 70%

To explore which subgroups might benefit from pre-CCRT feeding-stoma creation, patients were stratified by tumor-occupying proportion and tumor length. Among patients with tumor-occupying proportion ≥ 70%, feeding-stoma creation was associated with a lower incidence of post-CCRT cachexia (64.7% vs. 82.1%, *P* = 0.079), better treatment response (OR: 2.78, *P* = 0.028, Supplementary Table 3), as well as significantly longer median PFS (6 vs. 4 months, *P* = 0.012; Fig. 4a) and OS (11 vs. 9 months, *P* = 0.009; Fig. 4b). Baseline characteristics among patients with tumor-occupying proportion ≥ 70% are listed in Supplementary Table 4. After PSM, 68 patients with tumor-occupying proportion ≥ 70% were included in the matched feeding-stoma model, with 34 patients in the feeding-stoma (+) group and 34 patients in the feeding-stoma (-) group. After PSM, feeding-stoma creation was associated with a significantly longer median PFS (6 vs. 3 months, *P* = 0.004; Supplementary Fig.S3a) and OS (11 vs. 9 months, *P* = 0.006; Supplementary Fig. S3b). In order to investigate the relationship among feeding-stoma creation, treatment response, and overall survival, we performed Cox regression analysis of factors associated with overall survival (Supplementary Table 5). In univariate analysis, the feeding-stoma creation (hazard ratio (HR): 0.54, 95% CI: 0.33–0.88, *P* = 0.015) and a good treatment response (HR: 0.27, 95% CI: 0.16–0.46, *P* < 0.001) were associated with a longer overall survival, while only the good treatment response remained the independent factor in multivariate analysis (HR: 0.28, 95% CI: 0.16–0.50, *P* < 0.001).

In contrast, among patients with tumor-occupying proportion < 70%, feeding-stoma creation was not associated with the incidence of post-CCRT cachexia (52.1% vs. 59.2%, *P* = 0.534), treatment response (*P* = 0.591, Supplementary Table 3), PFS (Fig. 4c) or OS (Fig. 4d). When stratified by tumor length (≥ 6 cm vs. < 6 cm), feeding-stoma creation was not significantly associated with post-CCRT cachexia, PFS, or OS in patients with ESCC (Supplementary Fig. 2).

## Discussion

This study introduces a new concept of the tumor-occupying proportion in advanced ESCC and demonstrates its predictive value for post-CCRT cachexia. Our findings show that a tumor-occupying proportion ≥ 70% is strongly associated with more severe dysphagia, higher rates of post-CCRT cachexia, and poorer survival outcomes. Importantly, patients in this high-risk group were associated with a better prognosis with pre-CCRT feeding-stoma creation, showing a trend of reduction of post-CCRT cachexia, better treatment response and significant improvements in both progression-free and overall survival.

Previous researches have reported that routine feeding-stoma creation does not universally improve survival in patients with advanced esophageal cancer [14, 16, 22, 23]. However, our study refines this understanding by identifying a specific subgroup—those with tumor-occupying proportion ≥ 70%—who derive significant survival benefit from the intervention. This finding provides a targeted clinical indication for feeding-stoma creation, rather than recommending it for all patients.

Nutritional status is a critical determinant of cancer treatment tolerance. A poor nutritional status is closely linked to progression and worse outcomes of cancer [4, 24, 25]. Furthermore, malnutrition in ESCC patients may contribute to immune dysfunction, increased treatment-related complications, and a higher likelihood of CCRT discontinuation [26]. Our results highlight tumor-occupying proportion ≥ 70% as a strong predictor of post-CCRT cachexia, and was associated with worse treatment response, PFS and OS. These findings support consideration of early nutritional intervention through feeding-stoma in this high-risk group [16].

In our data, feeding-stoma creation was associated with a higher likelihood of favorable treatment response among patients with tumor-occupying proportion ≥ 70%, and multivariable models showed an association between feeding-stoma creation and treatment response, with treatment response in turn associated with survival. However, the mechanism underlying the observed survival benefit remains uncertain. The improved outcomes associated with feeding-stoma creation may be primarily mediated by improved nutritional status and treatment tolerance, which could reduce complications and enable completion of planned CCRT. Alternatively, the survival advantage may be indirectly mediated through enhanced CCRT efficacy, reflected by improved treatment response. Nonetheless, given the observational design, treatment response should be considered a potential mediator rather than an established mechanism, and residual confounding cannot be excluded.

In contrast, patients with tumor-occupying proportion < 70% are less likely to develop post-CCRT cachexia and, therefore, do not benefit from feeding-stoma. Although longer tumor length was also associated with post-CCRT cachexia, it was not linked to survival benefits from feeding-stoma creation. Given potential complications, such as mechanical bowel obstruction, infections, dislodgement [27, 28], and increased healthcare utilization [29], feeding-stoma placement should be reserved for those most likely to benefit. This individualized approach may prevent unnecessary interventions and improve quality of life [30].

This study has several limitations. First, its single-center design and relatively small sample size, particularly among female patients, may limit generalizability. In addition, patients receiving neoadjuvant CCRT followed by surgery were excluded to avoid treatment heterogeneity that could confound outcomes. Second, although higher tumor-occupying proportion was an independent predictor of post-CCRT cachexia in multivariate analysis, the effective size was relatively small (OR:1.03, 95% CI: 1.02–1.05, *P* = 0.003), which may lead to model overfitting. Further large scale, prospective study is warranted to confirm our finding. Third, details on CCRT dosing or compliance were not analyzed due to their complexity. Therefore, the underlying mechanism of feeding-stoma creation on improving survival remains speculative. Further studies are warranted to investigate whether feeding-stoma creation can improve CCRT dose-intensity, increase completion rates and decrease interruption rates. Fourth, transient weight loss related to acute treatment-related toxicity or short-term nutritional disturbances may occur during the CCRT, but recover after CCRT. Therefore, the interpretation of post-CCRT cachexia may be underestimate. Further prospective study with complete longitudinal nutritional assessments throughout the CCRT period is warranted. Fifth, tumor-occupying proportion may be influenced by luminal distension, endoscope position, and image selection. Although interobserver reliability was excellent, the absence of external validation limits generalizability. Future prospective study is needed for validation. Finally, mortality in ESCC patients arises from diverse, multifactorial causes, including tumor invasion, infection, cachexia, and treatment toxicity. While all may relate to ESCC to varying degrees, distinguishing direct cancer-attributable deaths from other causes remains challenging. Consequently, the survival outcomes may be overestimated.

## Conclusion

A higher tumor-occupying proportion (≥ 70%) is strongly associated with post-CCRT cachexia and poor survival outcomes in advanced ESCC. However, patients in this subgroup benefit significantly from pretreatment feeding-stoma, which is associated with improved PFS and OS. These findings suggest that tumor-occupying proportion can serve as a useful criterion for selecting candidates for pre-CCRT feeding-stoma creation, optimizing nutritional support, and improving clinical outcomes.

## Supplementary Information

Below is the link to the electronic supplementary material.

Supplementary file1 (DOCX 980 KB)
