# Supplementary material for: Pretreatment feeding‑stoma placement in advanced esophageal cancer: defining optimal patient selection criteria
Source: Esophagus. 2026 May 21;23(3):475–85. doi: 10.1007/s10388-026-01210-6 (PMC13319426; doi:10.1007/s10388-026-01210-6)
Supplement: Supplementary file 1 — Supplementary file1 (DOCX 980 KB) [file 10388_2026_1210_MOESM1_ESM.docx]

**Supplementary Table 1**. Patients’ characteristics associated with tumor-occupying proportion

|  | Tumor-occupying proportion < 70 %  (N = 170) | Tumor-occupying proportion ≥ 70 %  (N = 90) | *P*-value |
| --- | --- | --- | --- |
| Age (years) | 58.3 ± 10.5 | 57.3 ± 8.7 | 0.455 |
| Gender (M: F) | 161: 9 | 88: 2 | 0.339 |
| Body weight (kg) | 61.0 ± 11.5 | 58.7 ± 10.6 | 0.125 |
| BMI (kg/m^2^) | 22.1 ± 3.6 | 21.5 ± 3.4 | 0.176 |
| Albumin (g/dL) | 4.2 ± 0.4 | 4.0 ± 0.4 | 0.061 |
| Prealbumin (mg/dL) | 22.8 ± 7.0 | 19.4 ± 6.7 | 0.002 |
| Transferrin (mg/dL) | 221 ± 52 | 212 ± 54 | 0.290 |
| Dysphagia score (median, IQR, 25^th^-75^th^ percentile) | 1 (0 – 2) | 2 (2 – 3) | 0.001 |
| Cancer stage (I: II: III: IV) | 5: 10: 50: 78 | 0: 1: 22: 46 | 0.102 |
| T stage (T1: T2: T3) | 13: 18: 139 | 0: 3: 87 | 0.002 |
| N stage (N0: N1: N2: N3) | 12: 47: 52: 59 | 2: 30: 12: 45 | 0.121 |
| M stage (M0: M1) | 120: 50 | 48: 42 | 0.131 |
| Endoscopy findings |  |  |  |
| Tumor length (cm) | 5.2 ± 2.3 | 6.7 ± 3.0 | < 0.001 |
| Tumor thickness (cm) | 1.5 ± 1.0 | 1.6 ± 0.5 | 0.287 |

**Supplementary Table 2**. Patients’ characteristics associated with tumor length

|  | Tumor length < 6cm  (N = 148) | Tumor- length ≥ 6cm  (N = 112) | *P*-value |
| --- | --- | --- | --- |
| Age (years) | 57.6 ± 10.3 | 58.4 ± 9.3 | 0.494 |
| Gender (M: F) | 142: 6 | 107: 5 | 0.871 |
| Body weight (kg) | 60.3 ± 11.1 | 60.0 ± 11.4 | 0.820 |
| BMI (kg/m^2^) | 21.9 ± 3.6 | 21.9 ± 3.5 | 0.869 |
| Albumin (g/dL) | 4.2 ± 0.4 | 4.1 ± 0.4 | 0.217 |
| Prealbumin (mg/dL) | 21.9 ± 7.0 | 20.9 ± 7.1 | 0.367 |
| Transferrin (mg/dL) | 221 ± 52 | 212 ± 54 | 0.290 |
| Dysphagia score (median, IQR, 25^th^-75^th^ percentile) | 2 (0 – 2) | 2 (1 – 2) | 0.066 |
| Cancer stage (I: II: III: IV) | 5: 10: 45: 67 | 0: 1: 27: 57 | 0.017 |
| T stage (T1: T2: T3) | 11: 16: 121 | 2: 5: 105 | 0.015 |
| N stage (N0: N1: N2: N3) | 12: 40: 49: 46 | 2: 37: 15: 58 | 0.026 |
| M stage (M0: M1) | 100: 48 | 68: 44 | 0.295 |
| Endoscopy findings |  |  |  |
| Tumor-occupying portion (%) | 53.8 ± 22.7 | 65.5 ± 19.7 | < 0.001 |

**Supplementary Table 3**. The relationship between feeding-stoma creation and treatment response

| N = 260 | Feeding stoma (+)  (N = 62) | Feeding stoma (-)  (N = 198) | *P*-value |
| --- | --- | --- | --- |
| Treatment response |  |  | 0.155 |
| Complete response (CR) | 2 (3.2) | 22 (11.1) |  |
| Partial response (PR) | 35 (56.4) | 91 (45.9) |  |
| Stable disease (SD) | 10 (16.1) | 43 (21.7) |  |
| Progressive disease (PD) | 15 (24.1) | 42 (21.2) |  |
| Good response (CR+PR): Poor response (SD+PD) | 37: 25 | 113: 85 | 0.769 |

Patients with tumor-occupying proportion < 70 % (N = 170)

| N = 170 | Feeding stoma (+)  (N = 28) | Feeding stoma (-)  (N = 142) | *P*-value |
| --- | --- | --- | --- |
| Treatment response |  |  | 0.591 |
| Complete response | 2 (7.1) | 22 (15.4) |  |
| Partial response | 15 (53.5) | 72 (50.7) |  |
| Stable disease | 5 (17.8) | 28 (19.7) |  |
| Progressive disease | 6 (21.4) | 20 (14.0) |  |
| Good response (CR+PR): Poor response (SD+PD) | 17: 11 | 94: 48 | 0.665 |

Patients with tumor-occupying proportion ≥ 70 % (N = 90)

| N = 90 | Feeding stoma (+)  (N = 34) | Feeding stoma (-)  (N = 56) | *P*-value |
| --- | --- | --- | --- |
| Treatment response |  |  | 0.073 |
| Complete response | 0 | 0 |  |
| Partial response | 20 (58.8) | 19 (33.9) |  |
| Stable disease | 5 (14.7) | 15 (26.7) |  |
| Progressive disease | 9 (26.4) | 22 (39.2) |  |
| Good response (CR+PR): Poor response (SD+PD) | 20: 14 | 19: 37 | 0.028 |

**Supplementary table 4.** Baseline characteristics in patients with and without feeding-stoma creation among those with tumor-occupying proportion ≥ 70 %

|  | Feeding stoma (+)  (N = 34) | Feeding stoma (-)  (N = 56) | *P*-value |
| --- | --- | --- | --- |
| Age (years) | 58.3 ± 6.8 | 56.7 ± 9.6 | 0.429 |
| Gender (M: F) | 33: 1 | 55: 1 | 0.999 |
| Body weight (kg) | 56.4 ± 10.4 | 60.1 ± 10.5 | 0.110 |
| BMI (kg/m^2^) | 20.8 ± 3.8 | 21.9 ± 3.2 | 0.181 |
| Albumin (g/dL) | 3.9 ± 0.5 | 4.1 ± 0.3 | 0.144 |
| Prealbumin (mg/dL) | 18.3 ± 7.3 | 20.0 ± 6.3 | 0.312 |
| Transferrin (mg/dL) | 197 ± 57 | 221 ± 52 | 0.079 |
| Dysphagia score (median, IQR, 25^th^-75^th^ percentile) | 2 (2 – 3) | 2 (2 – 3) | 0.190 |
| Cancer stage (I: II: III: IV) | 0: 0: 12: 22 | 0: 1: 13: 42 | 0.383 |
| T stage (T1: T2: T3) | 0: 1: 33 | 0: 2: 54 | 0.999 |
| N stage (N0: N1: N2: N3) | 1: 7: 7: 18 | 1: 23: 5: 27 | 0.133 |
| M stage (M0: M1) | 23: 11 | 25: 31 | 0.057 |
| Endoscopy findings |  |  |  |
| Tumor length (cm) | 6.9 ± 3.0 | 7.8 ± 3.1 | 0.100 |
| Tumor thickness (cm) | 2.4 ± 1.2 | 2.0 ± 0.7 | 0.069 |

**Supplementary table 5**. Cox regression analysis of factors associated with overall survival among patients with tumor-occupying proportion ≥ 70 % (N = 90)

| Factors | Univariate | | Multivariate | |
| --- | --- | --- | --- | --- |
|  | HR (95% CI) | *p* | HR (95% CI) | *p* |
| Age (years) | 1.00 (0.98-1.03) | 0.658 |  |  |
| Gender (male) | 0.59 (0.14-2.44) | 0.472 |  |  |
| BMI (kg/m^2^) | 0.97 (0.91-1.04) | 0.516 |  |  |
| Albumin (g/dL) | 0.94 (0.56-1.58) | 0.842 |  |  |
| Prealbumin (mg/dL) | 1.00 (0.96-1.04) | 0.985 |  |  |
| Transferrin (mg/dL) | 1.00 (0.99-1.00) | 0.946 |  |  |
| Cancer stage | 1.58 (0.98-2.56) | 0.060 | 1.60 (0.99-2.58) | 0.051 |
| Feeding-stoma (+) | 0.54 (0.33-0.88) | 0.015 | 0.84 (0.49-1.45) | 0.552 |
| Treatment response (good) | 0.27 (0.16-0.46) | <0.001 | 0.28 (0.16-0.50) | <0.001 |

HR: hazard ratio

**
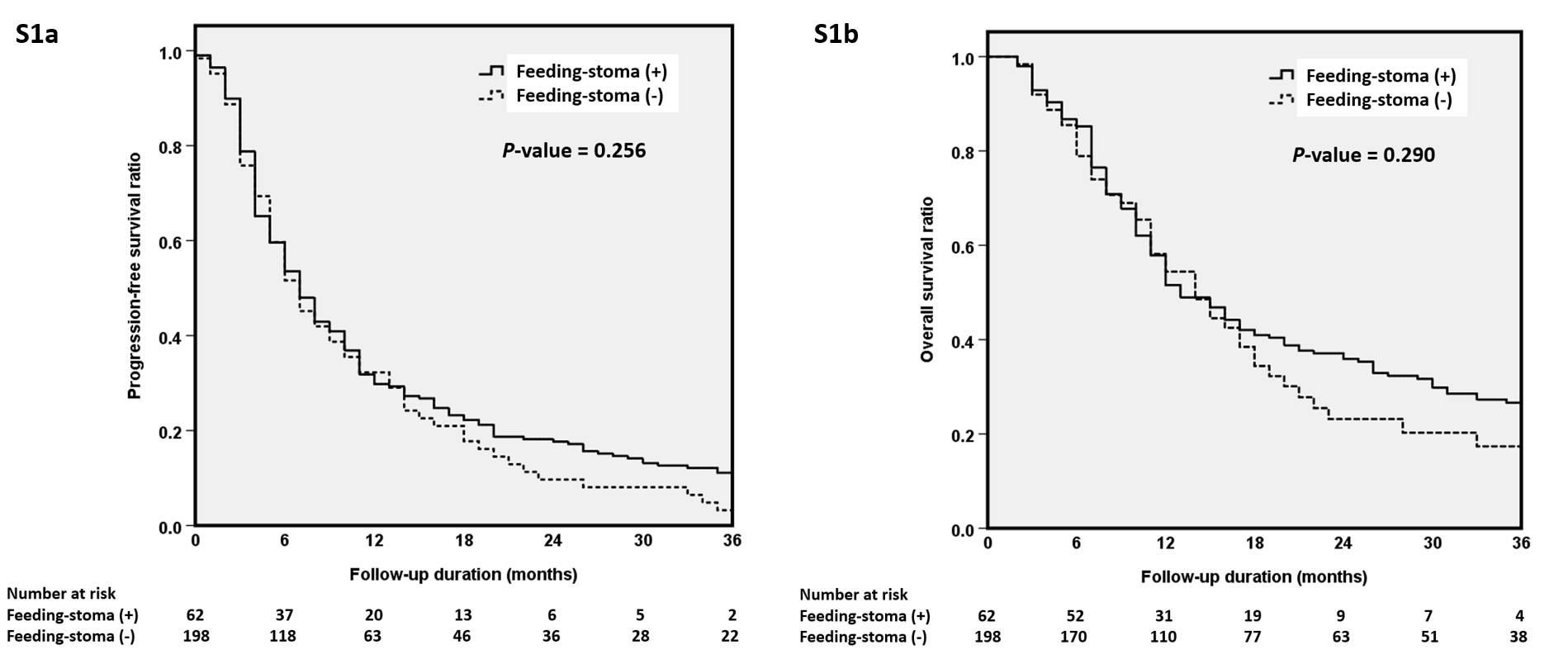
**

**Supplementary Figure 1**. **Comparison of survival between patients with and without feeding‑stoma creation in the whole cohort.** (a) Kaplan–Meier curve comparing PFS between patients with and without feeding-stoma creation. (b) Kaplan–Meier curve comparing OS between patients with and without feeding-stoma creation.

**
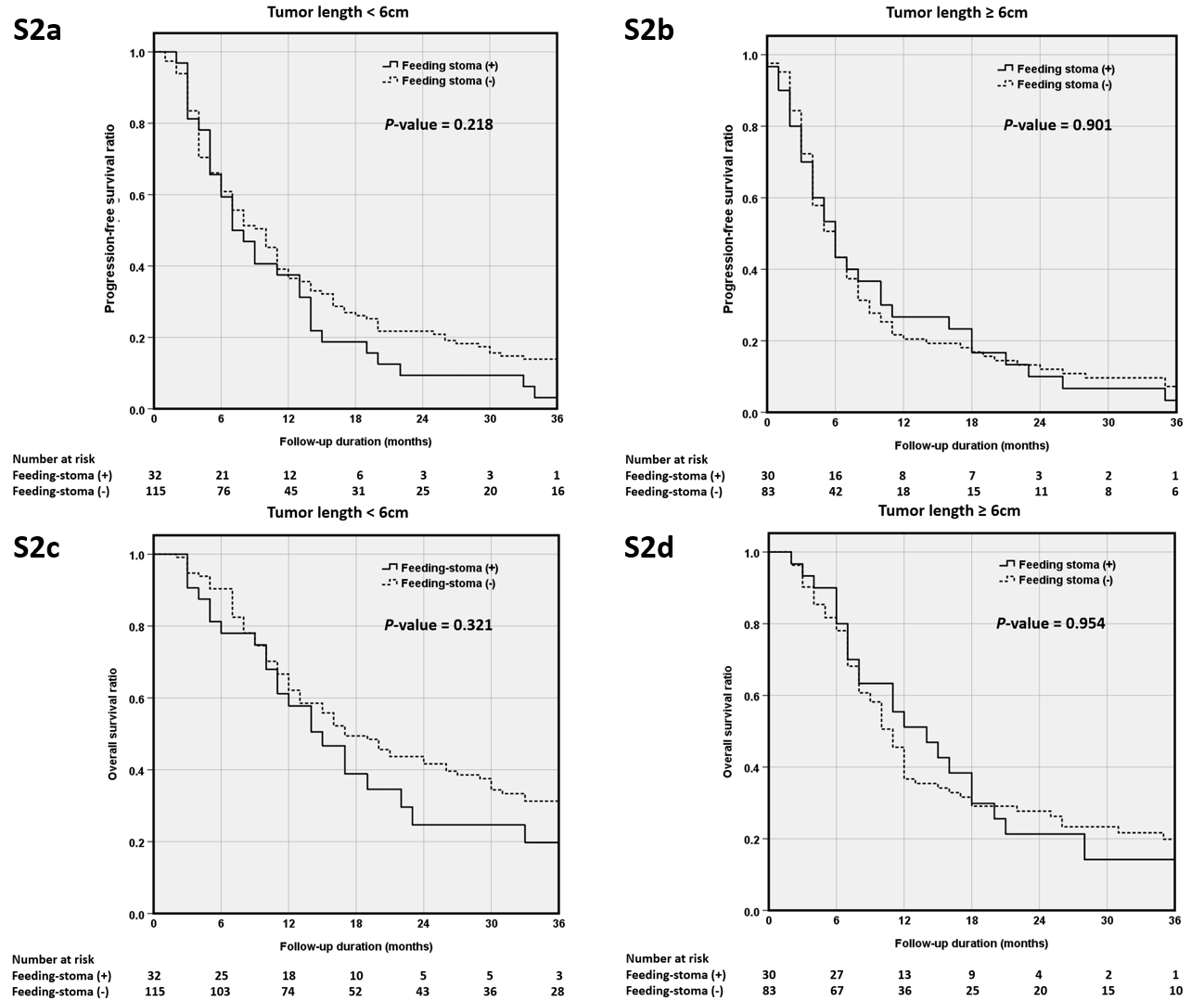
**

**Supplementary Figure 2**. **Comparison of survival between patients with and without feeding‑stoma creation, stratified by tumor length.** (a) PFS in patients with tumor length < 6 cm. (b) PFS in patients with tumor length ≥ 6 cm. (c) OS in patients with tumor length < 6 cm. (d) OS in patients with tumor length ≥ 6 cm.


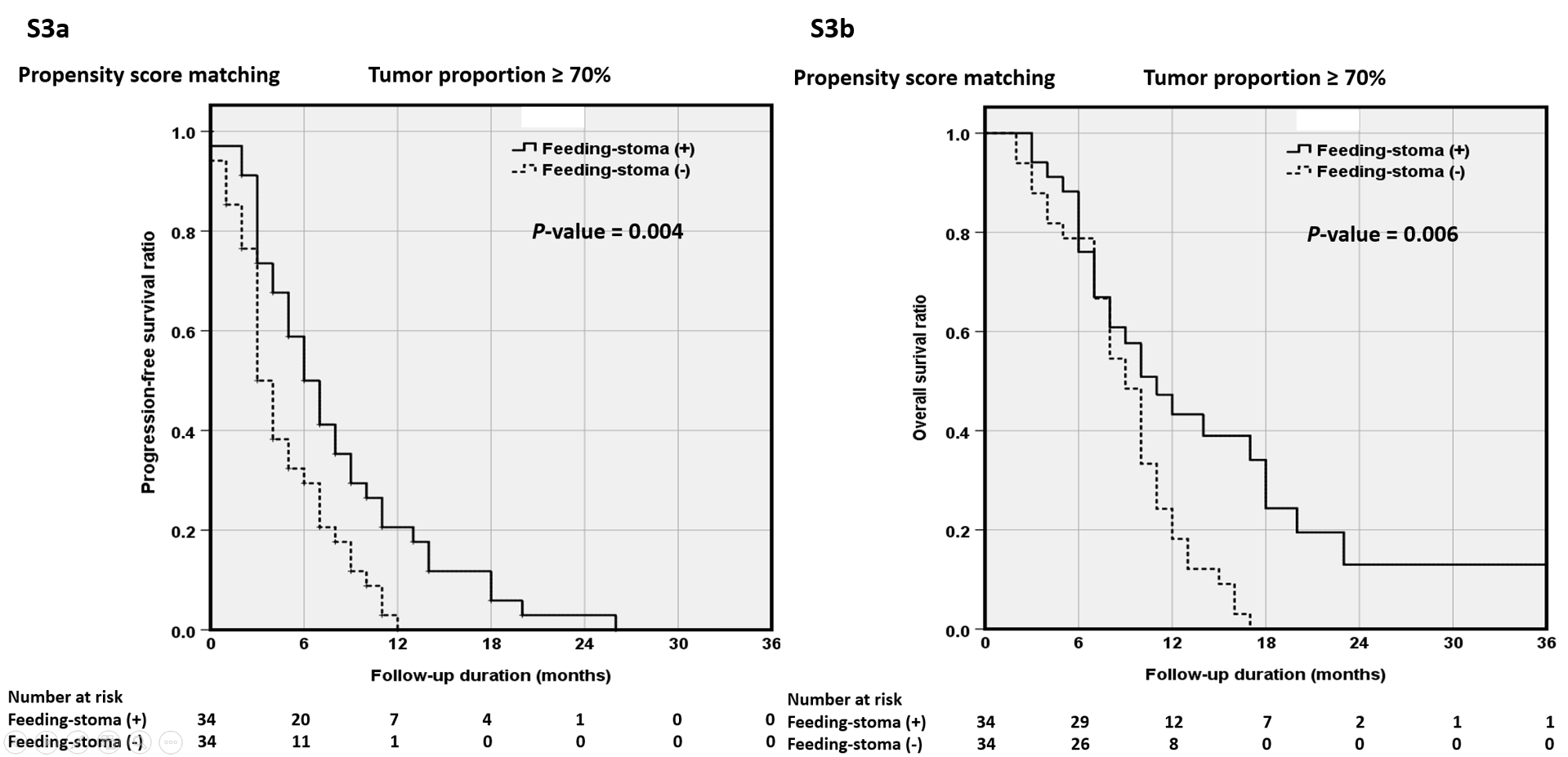


**Supplementary Figure 3**. **Comparison of survival between patients with and without feeding‑stoma creation among patients with tumor proportion over 70% via propensity score matching.** (a) Kaplan–Meier curve comparing PFS between patients with and without feeding-stoma creation. (b) Kaplan–Meier curve comparing OS between patients with and without feeding-stoma creation.

**
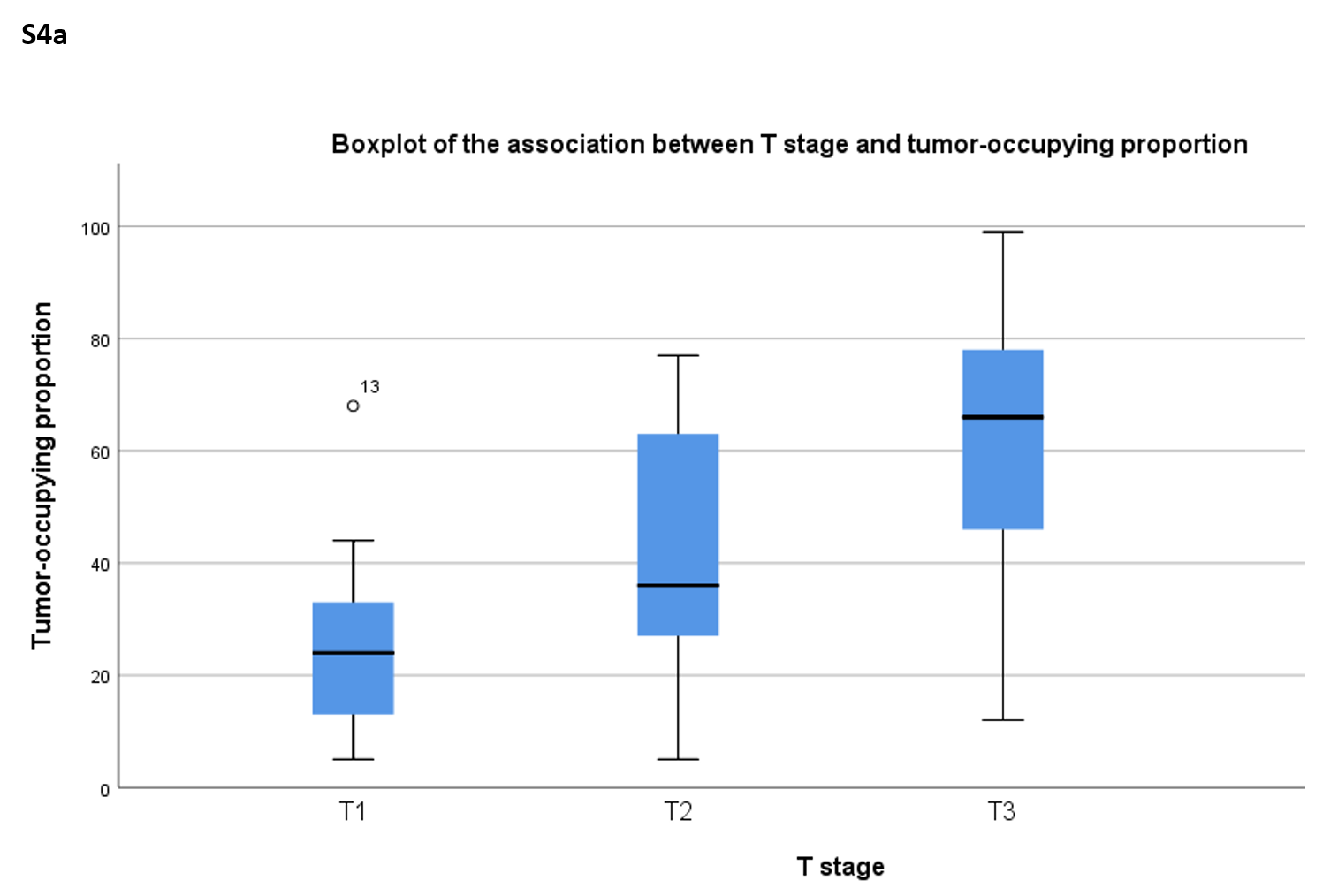
**

**
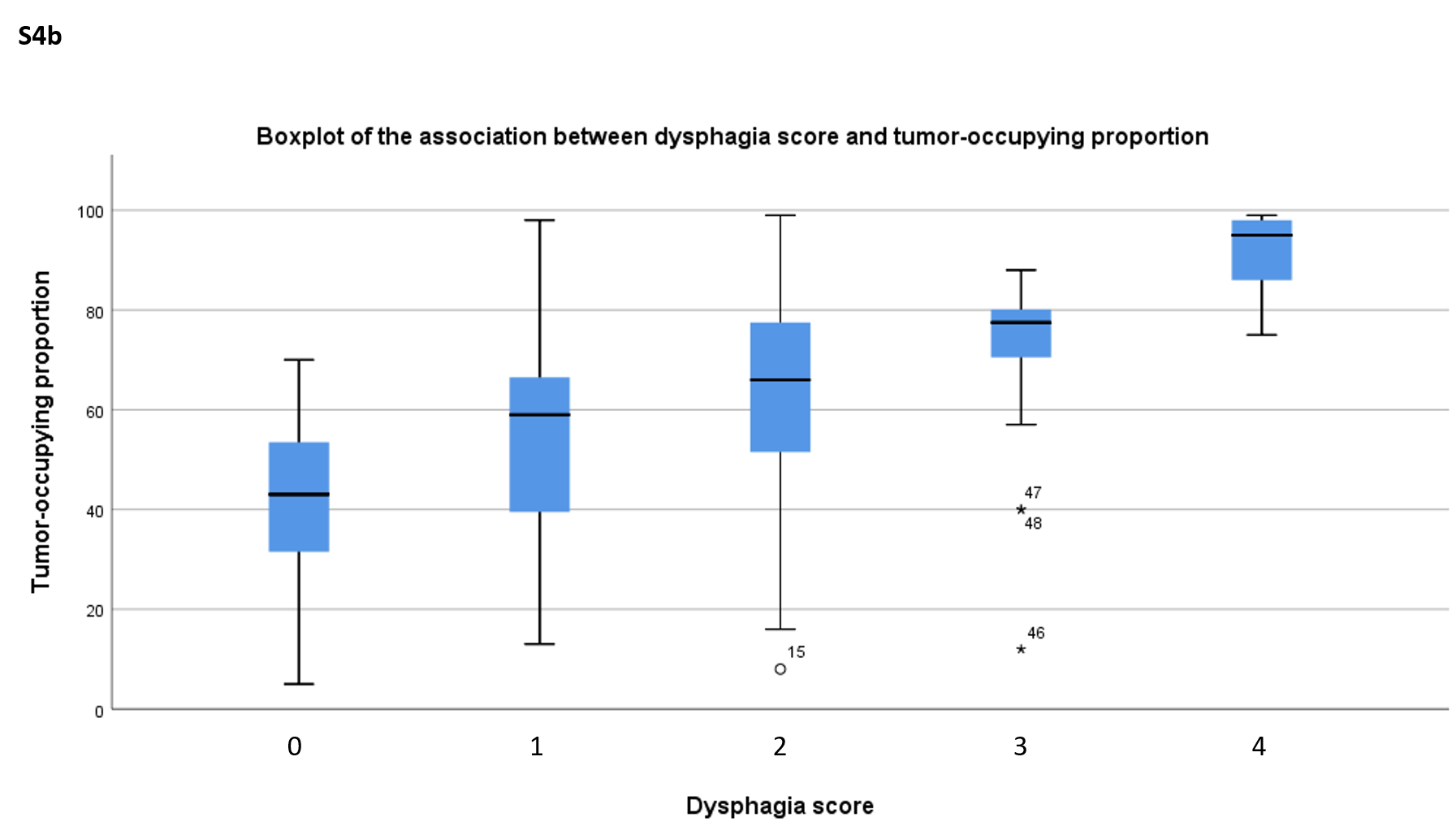
**

**Supplementary Figure 4. (a) The relationship between T staging and tumor-occupying proportion. (b) The relationship between dysphagia score and tumor-occupying proportion.**
